# Elevated Expression of Glycerol-3-Phosphate Phosphatase as a Biomarker of Poor Prognosis and Aggressive Prostate Cancer

**DOI:** 10.3390/cancers13061273

**Published:** 2021-03-13

**Authors:** Mohamed Amine Lounis, Veronique Ouellet, Benjamin Péant, Christine Caron, Zhenhong Li, Anfal Al-Mass, S. R. Murthy Madiraju, Anne-Marie Mes-Masson, Marc Prentki, Fred Saad

**Affiliations:** 1 Institut du Cancer de Montréal, Montreal, QC H2X 0A9, Canada; amine.lounis@umontreal.ca (M.A.L.); veronique.ouellet.chum@ssss.gouv.qc.ca (V.O.); benjamin.peant.chum@ssss.gouv.qc.ca (B.P.); christine.caron.chum@ssss.gouv.qc.ca (C.C.); anne-marie.mes-masson@umontreal.ca (A.-M.M.-M.); 2 Centre de Recherche du Centre Hospitalier de l’Université de Montréal (CRCHUM), Montreal, QC H2X 0A9, Canada; zhenhong919@gmail.com (Z.L.); anfal.al-mass@mail.mcgill.ca (A.A.-M.); murthy.madiraju@crchum.qc.ca (S.R.M.M.); 3 Department of Nutrition and Montreal Diabetes Research Center, CRCHUM, Montreal, QC H2X 0A9, Canada; 4 Département de Médecine, Université de Montréal, Montreal, QC H2X 0A9, Canada; 5 Département de Chirurgie, Université de Montréal, Montreal, QC H2X 0A9, Canada

**Keywords:** prostate cancer, predictive biomarker, G3PP, poor prognosis, metabolism, immunohistochemistry

## Abstract

**Simple Summary:**

A number of diseases, including cancers, can be diagnosed with “biomarkers”, such as specific proteins, hormones, or mutations in some genes. These molecules reflect abnormal processes in the affected organs, and are useful for diagnosis and disease treatment options. The need exists to have reliable markers for various types of cancers such as prostate cancer (PC). Many cancers show high utilization of glucose for their growth; we recently identified an enzyme, glycerol 3-phosphate phosphatase (G3PP), that can modulate glucose utilization by cells. Our work revealed a high expression of G3PP in prostate cancer cells in patients with aggressive tumors. With further validation, G3PP expression in prostate cancer tumors may become a useful prognostic biomarker and aid in the management of patients with PC.

**Abstract:**

The limitations of the biomarker prostate-specific antigen (PSA) necessitate the pursuit of biomarkers capable of better identifying high-risk prostate cancer (PC) patients in order to improve their therapeutic management and outcomes. Aggressive prostate tumors characteristically exhibit high rates of glycolysis and lipogenesis. Glycerol 3-phosphate phosphatase (G3PP), also known as phosphoglycolate phosphatase (PGP), is a recently identified mammalian enzyme, shown to play a role in the regulation of glucose metabolism, lipogenesis, lipolysis, and cellular nutrient-excess detoxification. We hypothesized that G3PP may relieve metabolic stress in cancer cells and assessed the association of its expression with PC patient prognosis. Using immunohistochemical staining, we assessed the epithelial expression of G3PP in two different radical prostatectomy (RP) cohorts with a total of 1797 patients, for whom information on biochemical recurrence (BCR), metastasis, and mortality was available. The association between biomarker expression, biochemical recurrence (BCR), bone metastasis, and prostate cancer-specific survival was established using log-rank and multivariable Cox regression analyses. High expression of G3PP in PC epithelial cells is associated with an increased risk of BCR, bone metastasis, and PC-specific mortality. Multivariate analysis revealed high G3PP expression in tumors as an independent predictor of BCR and bone metastasis development. High G3PP expression in tumors from patients eligible for prostatectomies is a new and independent prognostic biomarker of poor prognosis and aggressive PC for recurrence, bone metastasis, and mortality.

## 1. Introduction

Prostate cancer (PC) is the second-most diagnosed cancer in men and the fifth leading cause of death by cancer worldwide [1,2,3]. Although detection and treatments for PC have advanced in recent years, prostate-specific antigen (PSA) remains the only widely used biomarker for PC. Combining PSA with pathologic findings helps in stratifying patients in terms of risk. However, to optimize therapeutic decisions, there remains a need to identify biomarkers to better predict PC prognosis and aggressiveness [4]. Considering that the 5-year survival rate for men with metastasized prostate cancer decreases to 31% from nearly 100% for localized prostate cancer patients [5], it has become imperative to identify a biomarker that can reliably predict which patients with localized prostate cancer are at high risk of developing metastasis.

Proliferating PC cells exhibit higher demand for glucose and glutamine for their metabolic needs for membrane synthesis and cellular multiplication. In particular, elevated glycolysis has been considered to be a hallmark of cancer cells [6]. Recent evidence indicates that glycolysis and anaplerosis are not sufficient for providing the appropriate amount of energy and necessary building blocks for cellular growth. Cancer cells also exhibit an increase in lipogenesis from glucose-derived glycerol-3-phosphate for their survival and proliferation [7,8]. Chronic exposure of cancer cells to elevated concentrations of fatty acids and glucose induces glucolipotoxicity/metabolic stress, leading to cell death [9,10,11]. To circumvent this and survive under conditions of toxicity due to nutrient excess, cancer cells deploy metabolic detoxification mechanisms to prevent the accumulation of toxic metabolites. Our recent discovery of glycerol-3-phosphate phosphatase (G3PP) in mammalian cells [12] (protein name: G3PP, Uniprot ID: A6NDG6; gene name *pgp*; 2 phosphoglycolate phosphatase, Gene ID: 283871) adds another level of regulation of glucose and lipid metabolism, as G3PP hydrolyzes glycerol-3-phosphate (Gro3P), a critical metabolite at the crossroads of glucose and lipid metabolism. G3PP was initially described as phosphoglycolate phosphatase on the basis of sequence homology to plant and bacterial enzymes, but its actual physiological function in mammalian cells was obscure [13]. However, we found that this protein actually functions as a hydrolase of glycerol-3-phosphate in mammalian cells, both in vitro and in vivo, and has significant similarity to the glycerol-3-phosphatase enzymes from yeast and *Mycobacterium* sp. [12] and is therefore named glycerol-3-phosphate phosphatase (G3PP), which is accepted by several protein databases (Uniprot and NCBI-proteins), while the original gene name remains *PGP*. We accordingly refer to this protein as G3PP throughout this manuscript. We have shown that increasing G3PP activity in rat hepatocytes decreases the synthesis of glycerolipids, glycolysis, and gluconeogenesis and protects the tumor-derived INS-1(832/13) pancreatic *β*-cell line from glucolipotoxicity [12]. On the other hand, RNAi-knockdown of G3PP increases the susceptibility to glucolipotoxicity in INS-1(832/13) cells and enhances glycolysis and glycerolipid synthesis in hepatocytes [12,13].

Based on the major role of G3PP in the regulation of glycolysis and lipid metabolism, it is conceivable that this enzyme plays a critical role in tumor cell metabolism and could be a potentially useful biomarker of PC aggressiveness.

In the present study, we investigated the expression of the G3PP protein as an independent predictor of biochemical recurrence (BCR), bone metastases, and PC-specific death in two independent cohorts.

## 2. Materials and Methods

### 2.1. Patients and Tissue Micro Array Series

Two retrospective cohorts of patients were used to create two different tissue micro array (TMA) series. The TF123-TMA cohort was composed of 285 treatment-naïve PC patients who signed an informed consent form to participate in the Centre de recherche du Centre hospitalier de l’Université de Montréal (CRCHUM) PC biobank, affiliated with the Cancer Research Network (RRCancer) [14,15,16,17]. These patients underwent radical prostatectomy (RP) procedures between 1992 and 2006 at the Centre hospitalier de l’Université de Montréal (CHUM). Ethics approval for biobanking and evaluation of biomarkers was obtained from the Comité d’éthique de la recherche du CRCHUM. The Canadian Prostate Cancer Biomarker Network (CPCBN)-TMA series was composed of treatment-naïve specimens from 1512 patients who underwent RP between 1990 and 2011. These patient samples came from five biobanks: CRCHUM, Research Institute of the McGill University Health Center (RI-MUHC), Centre de recherche du Centre hospitalier Universitaire de Québec–Université Laval (CRCHUQ–UL), University Health Network (UHN) of Toronto, and Vancouver Prostate Centre (VPC). All patients signed an informed consent for the use of their prostate tissue samples in research. The local ethics review board approved the inclusion of specimens into the CPCBN multicenter resource (www.tfri.ca/cpcbn, accessed on: 20 June 2019).

A pathologist reviewed hematoxylin/eosin (H&E)-stained slides from archived formalin-fixed paraffin-embedded (FFPE) RP tissue blocks and circled the areas of interest. For each patient, 0.6 mm cores (2–4 cores of tumor tissue and 1–2 cores of benign tissue) were taken from the FFPE blocks and arrayed on receiver blocks.

### 2.2. Immunohistochemistry

We confirmed the specificity of the G3PP antibody using immunohistochemistry (IHC) and Western blot in prostate cancer cell lines and transfected prostate cell lines with vector or G3PP pcDNA (overexpression of G3PP) (Figure S1A). Immunohistochemistry assays for TMAs were performed on 4 µm sections of each TMA block using the Benchmark XT autostainer (Ventana Medical Systems, Tucson, AZ, USA). Sections were subjected to antigen retrieval in Cell Conditioning 1 (#950-124, Ventana Medical System) for 90 min, stained using pre-diluted anti-G3PP mouse monoclonal antibody (1:100, sc-390883 (E-10), Santa Cruz Biotechnology, Santa Cruz, CA, USA), and then manually added to the slides and incubated at 37°C for 60 min. Antibody binding was revealed using the UltraView universal DAB detection kit (#760-500, Ventana Medical Systems). Counterstaining was achieved using hematoxylin and bluing reagents (#760-2021 and #760-2037, Ventana Medical Systems). Tissues were dehydrated and mounted using SubX mounting media (Leica microsystems, Concord, ON, Canada). Isotype control (Mouse IgG2a (Cat number: 11-4724-81), Invitrogen, California, CA, USA) and secondary antibody only were used as negative controls for immunohistochemistry (IHC). All sections were scanned using a VS-110 microscope with a 20× 0.75 NA objective and a resolution of 0.3225 µm (Olympus Canada Inc., Richmond Hill, ON, Canada).

Scanned images were imported into VisiomorphDP software (Visiopharm, Hørsholm, Denmark). Semi-automated analysis protocol packages (APPs) were established to determine the mean intensity (MI) of G3PP, specifically in the epithelium (Figure S1B). Cores with less than 5% epithelial cells or damage were discarded (less than 1%). G3PP antibody was validated using tissues from liver-specific G3PP knockout mice. Absence of G3PP protein expression in the hepatocytes of liver-specific G3PP-KO mice and unaltered expression in other organs (brain, heart, skeletal muscle, and adipose tissues) with appropriate loading controls were verified in Western blots using the G3PP antibody (Figure S1). We also added a Western blot showing G3PP expression in two normal prostate cells (RWPE and PZ-HPV7) and four prostate cancer cell lines (LNCaP, PC3, 22RV1, and DU145) as this is relevant to the present study (Figure S1).

### 2.3. Statistical Analyses

Statistical analyses were performed with SPSS software 25.0 (SPSS Inc., Chicago, IL, USA). The correlation with clinicopathological variables was estimated with a nonparametric Spearman’s correlation test. The plan of analysis was to evaluate the association of G3PP expression with PC patient clinical endpoints, which included BCR, the development of bone metastasis, and PC-specific mortality. The cut-off applied for dichotomization of the data was defined by the 75th percentile of G3PP expression. BCR (defined as PSA at 0.2 ng/mL and rising with a decision for additional treatment, or PSA that did not achieve a level of <0.2 ng/mL following surgery defined as failed RP) [18], bone metastasis-free survival, and PC-specific survival curves were plotted using the Kaplan–Meier estimator, and the log-rank test was used to evaluate significant differences. The univariate and multivariate proportional hazard models (Cox regression) were used to estimate the hazard ratios (HRs) for G3PP expression. In the rare instance where clinical data were missing, the case was withdrawn from the analyses. Box-plots were used to graphically represent G3PP protein expression level in core prostate cancer cells with different Gleason grades and adjacent benign tissue. The box represents the interquartile (IQ) range (Q1–Q3). These were coupled with two-tailed Mann–Whitney U tests for benign vs. tumor analysis and two-tailed Kruskal–Wallis tests for Gleason grade. Results were considered statistically significant at *p*-values < 0.05.

## 3. Results

### 3.1. Elevated Epithelial Expression of G3PP Is Associated with PC Aggressiveness

We first studied the expression of G3PP in the TF123 cohort and then examined the CPCBN-TMA series [14,15,16,17]. Clinicopathological characteristics of PC patients in each cohort are presented in Table 1.

**Figure 1:**
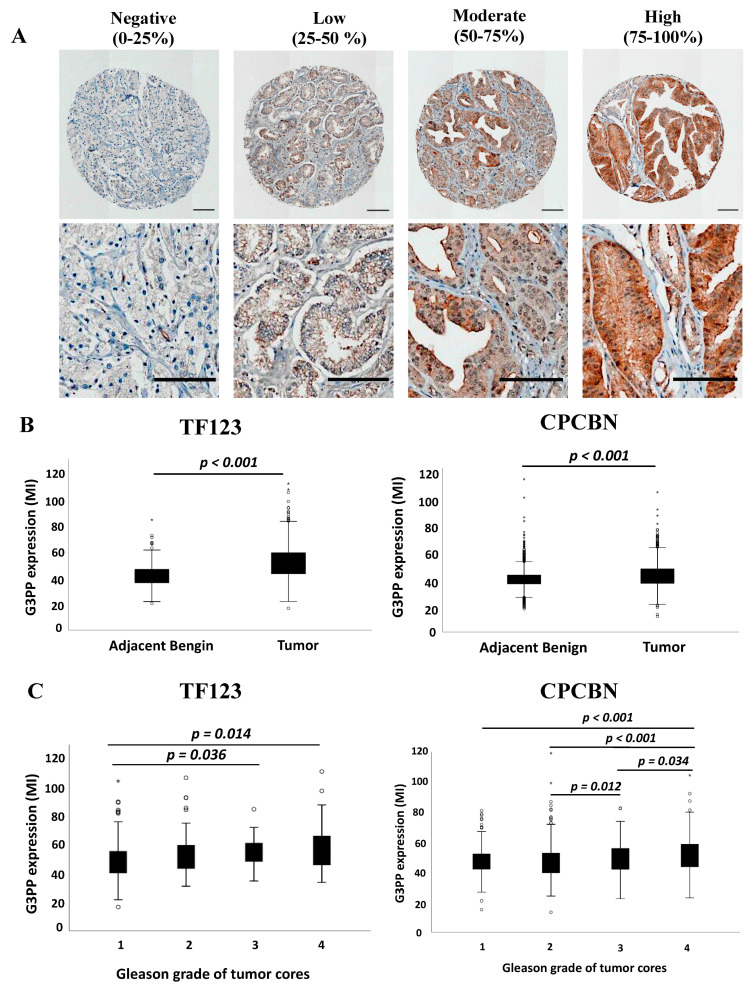
Epithelial expression of glycerol 3-phosphate phosphatase (G3PP) in prostate cancer tissues. (**A**) Representative images of G3PP immunostaining on tissue micro array (TMA) cores of prostate cancer. Images represent expression of G3PP protein at less than the 25th percentile, between the 25th and 50th, between 50th and 75th, and more than 75th percentile. Scale bars, 200 μm (top) and 50 μm (bottom). (**B**) Box-plot representation of epithelial expression of G3PP in prostate cancer tissues versus benign adjacent tissues of the TF123 and CPCBN tissue micro arrays (TMAs) (*p*-value, Mann–Whitney U test) and (**C**) box-plot representation of epithelial expression of G3PP in prostate cancer tissues in the different Gleason score categories (1, 2, 3, or 4) (*p*-value, Kruskal–Wallis test).

In adjacent benign and tumor tissue cores, G3PP was observed in the cytoplasm of epithelial cells while absent from the stroma (Figure 1A and Figure S1). Immunohistochemical staining of G3PP was homogeneous in the majority of tumor cores in terms of expression intensity. The mean intensity (MI) of G3PP varied between 28.6 and 109.5 for TF123-TMA and between 13.3 and 119.12 for CPCBN-TMA. We have categorized the expression of G3PP in the TMA samples on the basis of its immunoreactivity in IHC in different percentiles. Representative images of negligible, less than 25th percentile (0–25%); low, between 25th and 50th percentile (25–50%); moderate, between 50th and 75th percentile (50–75%); and high, more than 75th percentile (>75%) expression are presented in Figure 1A. G3PP expression was higher in tumor tissue cores compared to the benign adjacent cores (*p* < 0.001) (Figure 1B). Its expression also increased with Gleason grade group (Figure 1C).

### 3.2. High Expression of G3PP Is Associated with an Increased Risk of Biochemical Recurrence of PC

Correlations of epithelial G3PP expression in tumor cores with clinical characteristics were assessed on continuous and dichotomized data. We used the 75th percentile of G3PP (64.77 MI for TF123-TMA and 53.84 MI for CPCBN-TMA) as the threshold to dichotomize data based on the quartile method [19]. Expression of G3PP is considered low when ≤75% and high when >75%. Using dichotomization, we identified 66 (21.05%) PC patients with high expression of G3PP and 201 patients with low expression in the TF123 series. In the CPCBN-TMA series, 366 (24.20%) PC patients presented high expression of G3PP and 1098 patients presented with low expression.

**Figure 2:**
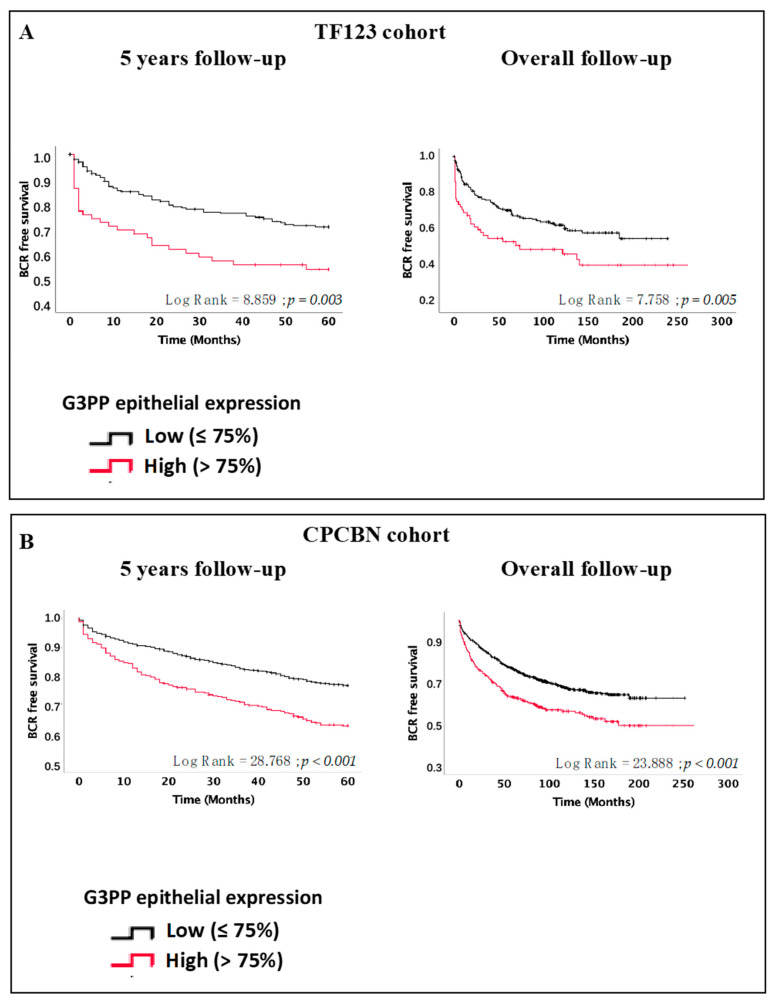
High expression of G3PP is associated with increased risk of biochemical recurrence within 5 years and overall follow-up. Kaplan–Meier biochemical recurrence (BCR)-free survival curves at 5 years and overall for the TF123 (**A**) and CPCBN (**B**) cohort TMAs.

Kaplan–Meier survival curves showed that high expression of G3PP was associated with an increased risk of BCR within 5 years in both the TF123 (log-rank = 8.859, *p* = 0.003) (Figure 2A, left panel) and the CPCBN-TMA series (log-rank = 28.768, *p* < 0.001) (Figure 2B, left panel). Interestingly, these results were also confirmed in an overall follow-up for both the TF123 (log-rank = 7.758, *p* = 0.005) (Figure 2A, right panel) and CPCBN-TMA series (log-rank = 23.888, *p* < 0.001) (Figure 2B, right panel).

Univariate Cox regression analyses for 5 years and overall follow-up demonstrated that all known predictors of BCR were statistically significant in both cohorts (Table 2 and Table S1). Univariate regression analyses of both continuous (HR 1.024 (95% CI 1.008–1.039), *p* = 0.003) and dichotomized (HR 1.932 (95% CI 0.854–2.162), *p* = 0.004) values of G3PP expression were associated with an increased risk of BCR in the TF123 series (Table 2). These results were validated in the CPCBN series in continuous (HR 1.031 (95% CI 1.022–1.039), *p* < 0.001) and dichotomized G3PP (HR 1.761 (95% CI 1.427-2.173), *p* < 0.001) (Table 2). When associated with the clinical parameters of BCR, epithelial G3PP expression remained an independent parameter, as indicated by the multivariate Cox regression analyses (Table 2). We also observed similar results in both cohorts after an overall follow-up (Table S1).

### 3.3. High Expression of G3PP Is a Predictor of Bone Metastasis within 10 Years

**Figure 3:**
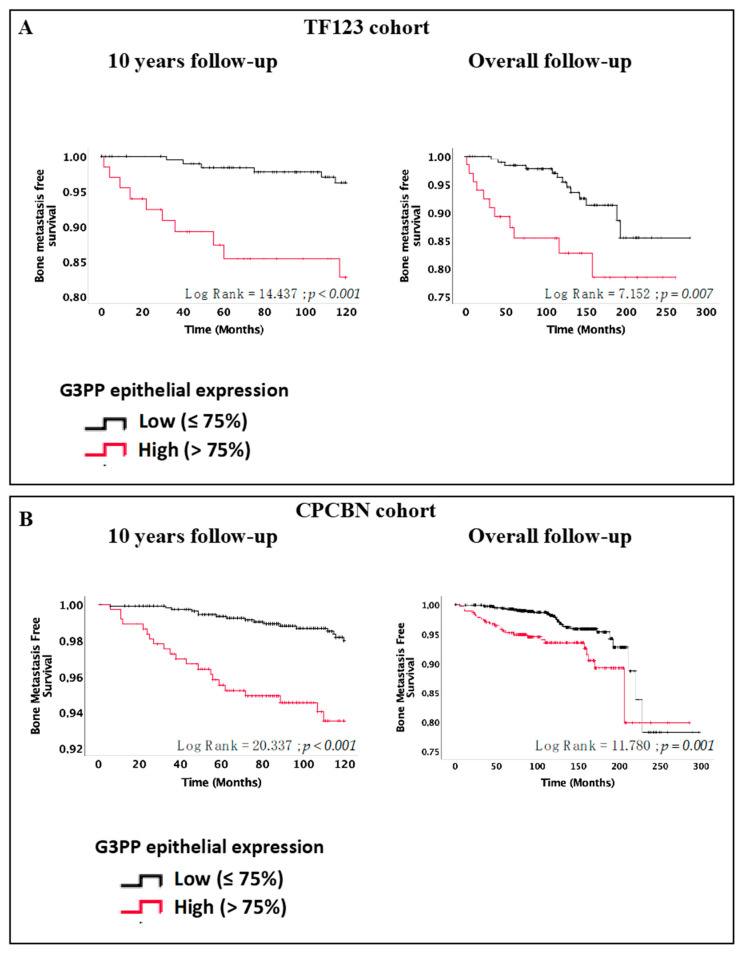
High expression of G3PP is associated with increased risk of bone metastases after 10 years and overall follow-up. Kaplan–Meier bone metastasis-free survival curves after 10 years and overall follow-up for the TF123 (A) and CPCBN (B) cohort TMAs.

Bone is the main site of distant metastasis that represents a major clinical endpoint of PC [20]. Lack of metastasis is a strong predictor of PC survival [21,22]. Kaplan–Meier estimates demonstrated that bone metastasis-free survival was shorter for patients with high G3PP expression in both TMA series (log-rank = 14.437, *p* < 0.001 for TF123 and log-rank = 20.337, *p* < 0.001 for CPCBN) at 10 years (Figure 3A,B, left panels). Similar results were obtained for the overall follow-up in both the TF123 (log-rank = 7.152, *p* = 0.007) and CPCBN series (log-rank = 11.780, *p* = 0.001) (Figure 3A,B, right panels).

Multivariate analyses of the CPCBN cohort TMA showed that G3PP expression was significantly associated with development of bone metastases in continuous (HR 1.054 (95% CI 1.024–1.085), *p* < 0.001) and dichotomized data (HR 5.691 (95% CI 2.066–15.580), *p* = 0.001) within 10 years (Table 3) or overall survival (Table S2). The CPCBN-TMA series confirmed that high G3PP expression was a strong predictor of bone metastasis development within 10-year follow-up using both continuous (HR 1.052 (95% CI 1.032–1.0730), *p* < 0.001) and dichotomized (HR 3.910 (95% CI 2.062–7.412), *p* < 0.001) values. Moreover, G3PP association with bone metastasis development remained significant when clinical parameters (prostate-specific antigen (PSA), Pathalogical Tumor-Node-Metastasis (pTNM), and Gleason score) were included in the model (Table 3).

### 3.4. High Expression of G3PP Is a Predictor of PC-Specific Mortality

**Figure 4:**
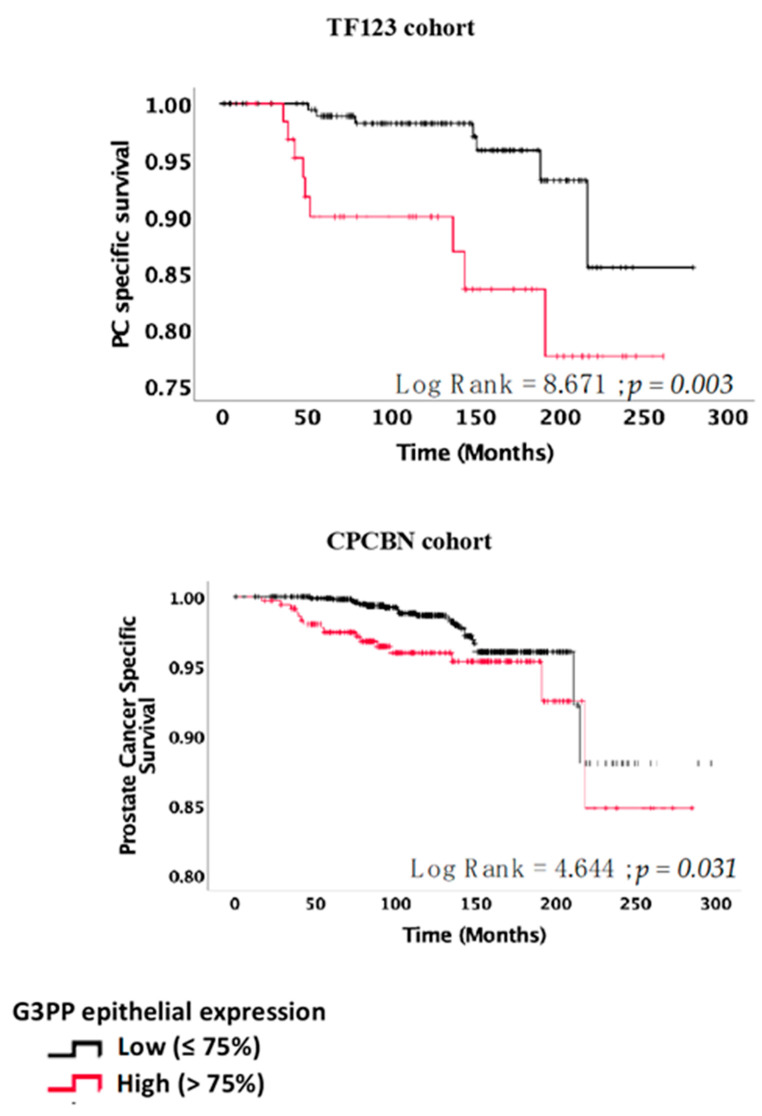
High expression of G3PP is associated with increased risk of prostate cancer-specific mortality. Kaplan–Meier bone metastasis-free survival curves after overall follow-up for the TF123 and CPCBN cohorts.

Using Kaplan–Meier estimators, we found that patients with high expression of G3PP showed reduced PC-specific survival. These results were observed in the TF123 cohort (log-rank = 8.671, *p* = 0.003) and were validated in the CPCBN (log-rank = 4.644, *p* = 0.031) TMA series (Figure 4).

The separation between low and high G3PP expression was particularly prominent within the first 5 years. Univariate Cox regression analyses showed a significant association of PC-specific mortality with continuous (HR 1.048 (95% CI 1.015–1.083), *p* = 0.004) and dichotomized (HR 3.990 (95% CI 1.477–10.774), *p* = 0.006) G3PP expression in the TF123 cohort (Table 4). These results were also recapitulated in the CPCBN series (continuous: HR 1.035 (95% CI 1.013–1.058), *p* = 0.002 and dichotomized: HR 1.995 (95% CI 1.051–3.786), *p* = 0.035) (Table 4).

## 4. Discussion

Identification of novel biomarkers that can help improve the stratification of patients with prostate cancer remains an important objective for optimal clinical management. Currently, serum PSA is the only biomarker used in screening, prognosis, and therapeutic response/progression for patients with PC [23,24]. Because of the limitations of PSA, identifying additional biomarkers able to predict disease aggressiveness and aid in therapeutic decisions may lead to better outcomes and reduced PC mortality. A number of additional PC biomarkers are now being explored [25].

Here, using two different cohorts, we have identified a promising intracellular biomarker, G3PP, the elevated expression of which is associated with poor PC prognosis, particularly within the first 5 years of diagnosis. Using immunohistochemistry to assess the expression of G3PP in PC TMAs, we analyzed the expression with digital image analysis software. Immunohistochemistry is routinely used in pathology, and its combination with digital pathology provides a powerful tool for the standardization of biomarker expression analysis and evaluation that can be readily translated into clinical practice [26,27]. We observed that G3PP was localized in the cytoplasm of epithelial cells in RP specimens from treatment-naïve PC patients and found that high G3PP expression was associated with an increased risk of early and overall BCR in the TF123 cohort of 285 patients and in the larger, multi-center CPCBN cohort of 1512 patients. We also showed that high expression of G3PP is an independent predictor of BCR (PSA > 0.2 ng/mL). Importantly, bone metastasis-free survival and PC-specific survival were shorter for patients with higher G3PP expression in cancer cells. Moreover, this marker remained independent when analyses were adjusted for pathological staging of the primary tumor and Gleason score at RP for bone metastasis after 10 years of follow-up. This finding is important given that metastasis has been established as a reliable surrogate for survival in localized prostate cancer [22].

Interestingly, Kaplan–Meier analysis for BCR, bone metastasis, and prostate cancer-specific survival showed that elevated expression of G3PP is a predictive biomarker of early relapse and poor prognosis. The adoption of G3PP as a predictive biomarker by clinicians could help considerably in the management and treatment of patients. Patients with high expression of G3PP are more susceptible to harboring aggressive PC and may be candidates for earlier and more aggressive therapy. Patients considered to be at very high risk in terms of their clinical, pathologic, and biomarker characteristics eventually may be considered for an early multimodal approach.

G3PP is an enzyme that regulates the cellular level of Gro3P, a critical intermediary at the crossroads of glucose and lipid metabolism [13]. G3PP is expressed in most human tissues, with highest expression in the testes as well as the prostate based on expression data posted in the Human Protein Atlas database (https://www.proteinatlas.org/ENSG00000184207-PGP/tissue, accessed on 11 February 2021). Cancer cells exhibit a high rate of glycolysis. Importantly, studies show that high expression of glycolytic markers is associated with poor prognosis of PC [28]. Glycolysis generates reducing equivalents (NADH) in the cytosol that are transferred to the mitochondrial electron transport chain via the redox Gro3P-dehydrogenase shuttle. This shuttle is a major contributor to reactive oxygen species (ROS) production [29]. Under conditions of accelerated glycolysis together with excess availability of glucose, there can be significant Gro3P shuttle-mediated production of ROS, which has toxic effects on cells. We previously proposed that elevated activity of G3PP, by hydrolyzing excess glucose-derived Gro3P, will generate less toxic glycerol and potentially dampen ROS-mediated detrimental effects [13]. In line with this explanation, elevated Gro3P-dehydrogenase has been observed in several tumors [30]. Thus, in prostate cancer, G3PP could play an important role in preventing elevated ROS production during glucose utilization within the interior of tumors and may also provide a glycerol substrate for cancer cells at the tumor-front as well as to the periprostatic adipose tissue. Besides modulating ROS, G3PP regulates the glycerolipid/free fatty acid (FFA) cycle (lipogenesis followed by lipolysis) that plays an important role in cancer cell growth [31,32]. We have previously shown that oleate promotes breast cancer cell growth via the FFA receptor FFAR1/GPR40 [33] and PI-3-kinase activation [34] and that upregulation of cellular triacylglycerol-FFA cycling by oleate is associated with long-term serum-free survival of human breast-cancer cells [35].

Based on these observations, we believe that in PC tumors, the observed high expression of G3PP, *per se*, is not a causative factor for tumorigenesis, but is a mechanism by which tumor cells may relieve metabolic stress with consequences on cell growth and survival.

## 5. Conclusions

In conclusion, we have demonstrated the prognostic value of epithelial G3PP protein expression in two cohorts of PC patients treated with radical prostatectomy. This study is the first to highlight the value of G3PP as an early predictor of patients at high risk of recurrence, bone metastases, and mortality. With further validation, the use of G3PP as an additional prognostic biomarker may eventually help clinicians in the management of early-stage prostate cancer.

## Figures and Tables

**Table 1:** Description of the TF123 and Canadian Prostate Cancer Biomarker Network (CPCBN) cohorts.

Parameters	TF123	CPCBN
Number of patients	285	1562
Mean age at diagnosis (years)	62	62
Median follow-up (months)	129	116.5
Biochemical recurrence	116	511
Bone metastasis	27	65
Castrate-resistant status	27	74
Presence of positive margins	95	509
RP Gleason score		
≤3 + 3	138	456
3 + 4	94	603
4 + 3	19	230
≥4 + 4	29	211
Undetermined	9	12
Biochemical recurrence type		
PSA > 0.2 ng/mL and rising	74	319
Failed RP	42	101
Pathological staging of the primary tumor		
pT2	201	959
pT3	75	530
pT4	9	23
Death		
Prostate cancer-specific	19	39
Other cause	30	136
Overall	49	176

**Table 2:** Univariate and multivariate Cox regression analyses predicting biochemical recurrence (BCR) in the TF123 and CPCBN cohort TMAs after 5-year follow-up.

Cox Regression	Univariate				Multivariate			
TMA Series	TF123		CPCBN		TF123		CPCBN	
Parameters	*p*-Value	HR (95% CI)	*p*-Value	HR (95% CI)	*p*-Value	HR (95% CI)	*p*-Value	HR (95% CI)
Preoperative PSA	<0.001	1.061 (1.033–1.089)	<0.001	1.033 (1.028–1.037)	0.057	1.035 (0.993–1.073)	<0.001	1.020 (1.013–1.026)
pTNM	<0.001	2.884 (2.133–3.900)	<0.001	3.114 (2.627–3.692)	0.014	1.652 (1.105–2.470)	<0.001	1.829 (1.629–2.053)
RP Gleason score	<0.001	1.852 (1.549–2.214)	<0.001	2.109 (1.918–2.320)	0.001	1.458 (1.171–1.816)	<0.001	1.771 (1.590–1.972)
Margin	<0.001	3.349 (2.216–5.062)	<0.001	2.628 (2.156-3.203)	<0.001	2.525 (1.576–4.047)	<0.001	1.839 (1.485–2.276)
Tumor Tissue								
G3PP continuous	0.003	1.024 (1.008–1.039)	<0.001	1.031 (1.022–1.039)	0.200	1.010 (0.995–1.027)	<0.001	1.019 (1.010–1.028)
G3PP dichotomized	0.004	1.932 (0.854–2.162)	<0.001	1.761 (1.427–2.173)	0.073	1.538 (0.960–2.465)	0.001	1.427 (1.146–1.776)

**Table 3:** Univariate and multivariate Cox regression analyses predicting bone metastasis development in the TF123 and CPCBN TMA cohorts after 10 years of follow-up.

Cox Regression	Univariate				Multivariate	
TMA Series	TF123		CPCBN		CPCBN	
Parameters	*p*-Value	HR (95.0% CI)	*p*-Value	HR (95.0% CI)	*p*-Value	HR (95.0% CI)
Preoperative PSA	<0.001	1.060 (1.033–1.087)	0.061	1.017 (0.999–1.036)	-	-
pTNM	<0.001	7.490 (3.830–14.647)	<0.001	5.674 (3.410–9.440)	0.026	1.931 (1.081–3.451)
RP Gleason score	<0.001	3.704 (2.304–5.955)	<0.001	4.099 (2.802–5.995)	<0.001	3.835 (2.442–6.020)
Margin	<0.001	3.803 (2.582–5.601)	0.488	1.241 (0.673–2.288)	-	-
Tumor Tissue						
G3PP continuous	<0.001	1.054 (1.024–1.085)	<0.001	1.052 (1.032–1.073)	<0.001	1.015 (1.008–1.023)
G3PP dichotomized	0.001	5.691 (2.066–15.680)	<0.001	3.910 (2.062–7.412)	0.007	1.320 (1.080–1.613)

**Table 4:** Univariate and multivariate Cox regression analyses predicting prostate cancer-specific mortality in the TF123 and CPCBN TMA cohorts after overall follow-up.

Cox Regression	Univariate				Multivariate	
TMA Series	TF123		CPCBN		CPCBN	
Parameters	*p*-Value	HR (95% CI)	*p*-Value	HR (95% CI)	*p*-Value	HR (95% CI)
Preoperative PSA	0.021	1.061 (1.009–1.116)	0.095	1.018 (0.997–1.040)	-	-
pTNM	<0.001	5.655 (2.928–10.923)	<0.001	3.582 (2.089–6.142)	0.226	1.436 (0.799–2.582)
RP Gleason score	<0.001	3.968 (2.395–6.573)	<0.001	3.453 (2.413–4.940)	<0.001	3.314 (2.246–4.890)
Margin	0.248	1.731 (0.682–4.391)	0.073	1.766 (0.949–3.285)	-	-
Tumor Tissue						
G3PP continuous	0.004	1.048 (1.015–1.083)	0.002	1.035 (1.013–1.058)	0.062	1.022 (0.997–1.046)
G3PP dichotomized	0.006	3.990 (1.477–10.774)	0.035	1.995 (1.051–3.786)	0.177	1.561 (0.818–2.978)

## Data Availability

The datasets used and/or analyzed during the current study are available from the corresponding author on reasonable request.

## Supplementary Materials

The following are available online at https://www.mdpi.com/2072-6694/13/6/1273/s1. Figure S1: Validation of G3PP antibody and tumor core analysis using Visiomorph software; Table S1: Univariate and multivariate Cox regression analyses predicting biochemical recurrence in the TF123 and CPCBN cohorts after overall follow-up; Table S2: Univariate and multivariate Cox regression analyses predicting bone metastasis development in the TF123 and CPCBN TMA cohorts after overall follow-up; and Table S3: Full list of members of the CPCBN group of authors.
