# Supplementary material for: Elevated Expression of Glycerol-3-Phosphate Phosphatase as a Biomarker of Poor Prognosis and Aggressive Prostate Cancer
Source: Cancers (Basel). 2021 Mar 13;13(6):1273. doi: 10.3390/cancers13061273 (PMC8000625; doi:10.3390/cancers13061273)
Supplement: Supplementary file 1 [file cancers-13-01273-s001.pdf]

cancers MDPI

Supplementary

Elevated Expression of Glycerol-3-Phosphate Phosphatase as a Biomarker of Poor Prognosis and Aggressive Prostate Cancer

Mohamed Amine Lounis, Veronique Ouellet, Benjamin Péant, Christine Caron, Zhenhong Li, Anfal Al-Mass, S.R Murthy Madiraju, Anne-Marie Mes-Masson, Marc Prentki and Fred Saad

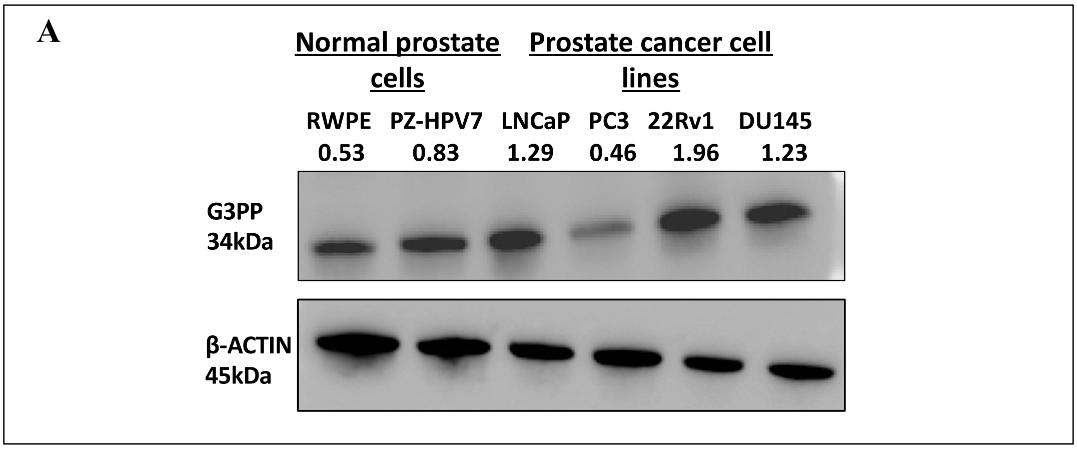

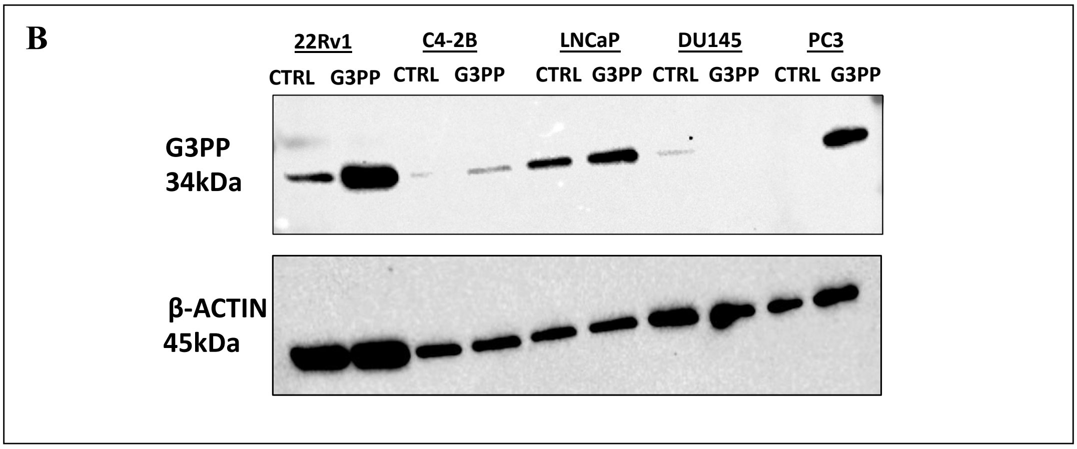

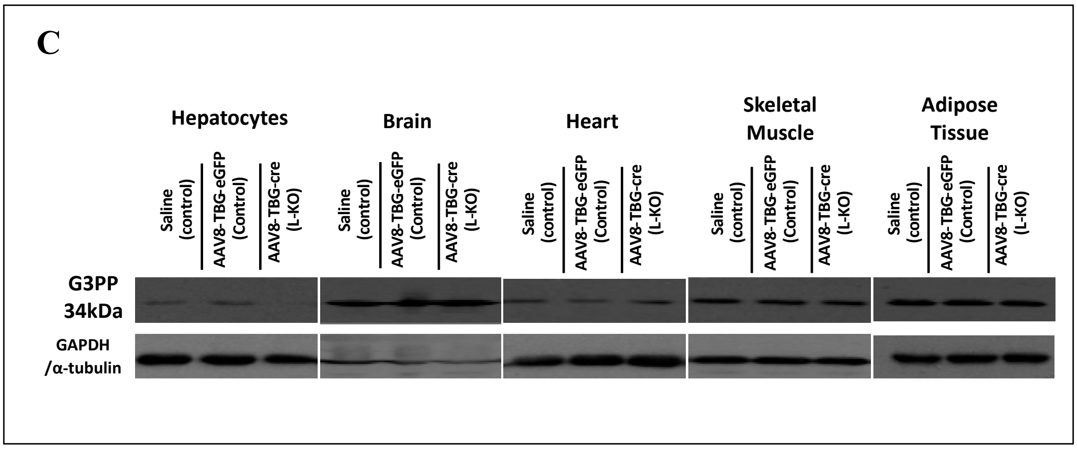

Figure S1. Validation of G3PP antibody by western blot analysis. (**A**) Western blot analysis of G3PP protein expression in two normal prostate epithelial cells (RWPE and PZ-HPV7) and four prostate cancer celllines (LNCaP, PC3, 22Rv1 and DU145) (**B**) Western blot analysis of prostate cancer cells transfected with Vector (CTR) or G3PP pcDNA G3PP (overexpression). (C) Liver specific deletion of G3PP/*PGP* using AAV8-TBG-cre virus, in vivo, in G3PP fl/fl mice. Male, 8 weeks old, G3PP flox/flox (fl/fl) mice fed standard chow diet (15% fat by energy; Harlan Teklad, Madison, WI, USA) received a single injection through the tail vein of AAV8-TBG-Cre (L-KO) or AAV8-TBG-eGFP (control) or only saline (control) (viral dose: 5×10^{10} Genome Copies/mice). After 4 weeks, the mice were sacrificed and different tissues were collected and hepatocytes were isolated from the livers by *in situ* collagenase (type XI; Sigma) perfusion. To validate G3PP deletion, protein was extracted from different tissues and hepatocytes and 40 µg of protein from each extract was used for western blot (primary Ab.: G3PP/PGP Antibody (E-10) (sc-390883) at dilution 1/1000; secondary Ab. m-IgGκ BP-HRP (sc-516102) at 1/10000 dilution). For all the tissues GAPDH was used as a housekeeping protein (loading control) except for the Adipose tissue for which α-tubulin was used. Results clearly demonstrate hepatocyte specific deletion of G3PP/PGP by the AAV8 mediated cre-recombinase expression. For all the normal and cancer cells β-actin was used as the housekeeping protein (loading control).

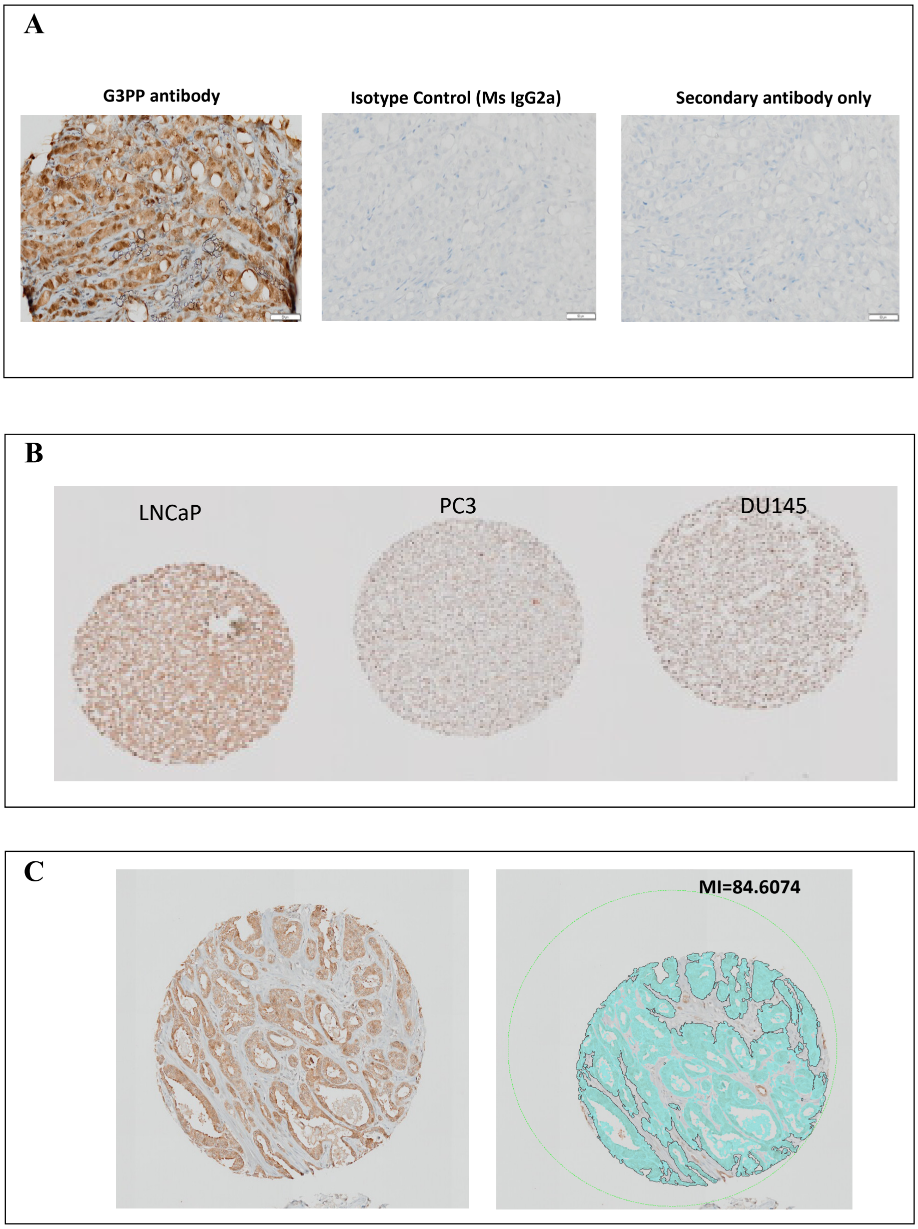

Figure S2. Validation of G3PP expression by immunohistochemistry and tumor core analysis using Visiomorph software. (A) Immunohistochemical staining of G3PP in human prostate cancer TMA. Representative images of Positive control (Staining with G3PP antibody; (E-10; sc-390883)) and negative controls (Mice Isotype IgG2a and absence of primary antibody). (B) G3PP

expression assessed by immunohistochemistry in three prostate cancer cell lines. (C) Determination of mean intensity of G3PP epithelial expression using semi-automated analysis protocol packages (APPs) of Visiomorph software. Representative image of IHC staining of G3PP (Left). Representative image of Visiomorph mask used to calculate Mean Intensity (MI) (Right)

Table 1. Univariate and multivariate Cox regression analyses predicting biochemical recurrence in the TF123 and CPCBN cohorts after overall follow-up.

**Cox Regression**	**Univariate**				**Multivariate**			
**TMA series**	**TF123**		**CPCBN**		**TF123**		**CPCBN**	
**Parameters**	**p-Value**	**HR (95% CI)**	**p-Value**	**HR (95% CI)**	**p-Value**	**HR (95% CI)**	**p-Value**	**HR (95% CI)**
Preoperative PSA	**<0.001**	1.064 (1.038-1.090)	**<0.001**	1.032 (1.028-1.037)	**0.011**	1.042 (1.009-1.075)	**<0.001**	1.020 (1.014-1.026)
pTNM	**<0.001**	1.810 (1.535-2.136)	**<0.001**	2.832 (2.432-3.298)	**0.001**	1.399 (1.140-1.718)	**<0.001**	1.590 (1.334-1.895)
RP Gleason score	**<0.001**	3.412 (2.349-4.958)	**<0.001**	1.955 (1.799-2.126)	**<0.001**	2.704 (1.772-4.126)	**<0.001**	1.682 (1.534-1.843)
Margin	**<0.001**	2.902 (2.194-3.837)	**<0.001**	2.353 (1.977-2.801)	**0.015**	1.598 (1.095-2.332)	**<0.001**	1.695 (1.410-2.038)
**Tumor Tissue**								
G3PP continuous	**0.003**	1.021 (1.007-1.036)	**<0.001**	1.025 (1.017-1.033)	0.142	1.011 (0.996-1.026)	**<0.001**	1.015 (1.008-1.023)
G3PP dichotomized	**0.006**	1.756 (1.172-2.631)	**<0.001**	1.593 (1.319-1.924)	**0.033**	1.592 (1.038-2.442)	**0.003**	1.342 (1.103-1.634)

Table S2. Univariate and multivariate Cox regression analyses predicting bone metastasis developement in the TF123 and CPCBN TMA cohorts after overall follow-up.

**Cox Regression**	**Univariate**				**Multivariate**			
**TMA series**	**TF123**		**CPCBN**		**TF123**		**CPCBN**	
**Parameters**	**p-Value**	**HR (95% CI)**	**p-Value**	**HR (95% CI)**	**p-Value**	**HR (95% CI)**	**p-Value**	**HR (95% CI)**
Preoperative PSA	**0.021**	1.061 (1.009-1.116)	**0.012**	1.019 (1.004-1.035)	0.283	(0.899-1.032)	0.836	0.998 (0.978-1.018)
pTNM	**<0.001**	5.679 (3.25-9.923)	**<0.001**	4.382 (2.864-6.706)	**0.005**	2.333 (1.292-4.214)	**0.006**	1.974 (1.219-3.195)
RP Gleason score	**<0.001**	3.508 (2.417-5.092)	**<0.001**	3.305 (2.516-4.341)	**<0.001**	2.977 (1.993-4.447)	**<0.001**	2.855 (2.145-3.800)
Margin	0.248	1.731 (0.682-4.391)	0.515	1.180 (0.717-1.940)	**-**	-	**-**	-
**Tumor Tissue**								
G3PP continuous	**0.046**	1.030 (1.000-1.060)	**<0.001**	1.034 (1.016-1.053)	0.146	1.026 (0.991-1.061)	0.077	1.018 (0.998-1.037)
G3PP dichotomized	**0.011**	2.856 (1.277-6.384)	**0.001**	2.379 (1.428-3.963)	**0.036**	2.567 (1.062-5.919)	**0.041**	1.727 (1.023-2.917)

Table 3. Full list of members of the CPCBN group of authors.

First Name	Initial	Last Name		Email	Affiliation
Fred		Saad	MD, FRCSC	fredsaad@videotron.ca	Département de Chirurgie, Université de Montréal, Centre de recherche du Centre hospitalier de l'Université de Montréal et Institut du cancer de Montréal
Anne-Marie		Mes-Masson	PhD, FCAHS, FRSC	anne-marie.mes-masson@umontreal.ca	Département de Médecine, Université de Montréal, Centre de recherche du Centre hospitalier de l'Université de Montréal et Institut du cancer de Montréal
Veronique		Ouellet	PhD	veronique.ouellet.chum@ssss.gouv.qc.ca	Centre de recherche du Centre hospitalier de l'Université de Montréal et Institut du cancer de Montréal
Dominique		Trudel	MD, FRCSC, PhD	dominique.trudel.chum@ssss.gouv.qc.ca	Département de pathologie et de biologie cellulaire, Université de Montréal, Centre de recherche du Centre hospitalier de l'Université de Montréal et Institut du cancer de Montréal et Institut du cancer de Montréal
Mathieu		Latour	MD, FRCSC	mathieu.latour.chum@ssss.gouv.qc.ca	Département de pathologie et de biologie cellulaire, Université de Montréal et Centre de recherche du Centre hospitalier de l'Université de Montréal
Veronique		Barrès	MSC	veronique.barres.chum@ssss.gouv.qc.ca	Centre de recherche du Centre hospitalier de l'Université de Montréal et Institut du cancer de Montréal
Nathalie		Delvoye	MSc	nathalie.delvoye.chum@ssss.gouv.qc.ca	Centre de recherche du Centre hospitalier de l'Université de Montréal et Institut du cancer de Montréal
Jean-Baptiste		Lattouf	MD, FRCSC	jean-baptiste.lattouf.chum@ssss.gouv.qc.ca	Centre de recherche du Centre hospitalier de l'Université de Montréal et Institut du cancer de Montréal
Pierre		Karakiewicz	MD, FRCSC	pierre.karakiewicz@umontreal.ca	Cancer Prognostics and Health Outcomes Unit, Centre hospitalier de l’Université de Montréal
Armen		Aprikian	MD, FRCSC	armen.aprikian@muhc.mcgill.ca	Deptartment of Surgery (Urology) McGill University and

					Cedars Cancer Centre and McGill University Health Centre
Fadi		Brimo	MD, FRCSC	fadi.brimo@mcgill.ca	Deptartment of Surgery (Urology) McGill University and McGill University Health Centre
Simone		Chevalier	PhD	simone.chevalier@mcgill.ca	Deptartment of Surgery (Urology) McGill University and McGill University Health Centre Research Institute
Lucie		Hamel	PhD	lucie.hamel@mail.mcgill.ca	McGill University Health Centre Research Institute
Eleonora		Scarlata	PhD	eleonora.scarlata@mail.mcgill.ca	McGill University Health Centre Research Institute
Louis		Lacombe	MD, FRCSC	louis.lacombe@crchudequebec.ulaval.ca	Centre de recherche du Centre hospitalier universitaire de Québec-Université Laval
Alain		Bergeron	PhD	alain.bergeron@crchudequebec.ulaval.ca	Centre de recherche du Centre hospitalier universitaire de Québec-Université Laval
Hélène		Hovington	BSc	helene.hovington@crchudequebec.ulaval.ca	Centre de recherche du Centre hospitalier universitaire de Québec-Université Laval
Hervé		Brisson	MSc	hervebrisson1959@gmail.com	Centre de recherche du Centre hospitalier universitaire de Québec-Université Laval
Céline		Veilleux	Inf.	celine.veilleux@crchudequebec.ulaval.ca	Centre de recherche du Centre hospitalier universitaire de Québec-Université Laval
Neil	E	Fleshner	MD, FRCSC	Neil.Fleshner@uhn.on.ca	Division of Urology, University of Toronto, Princess Margaret Hospital
Theodorus		van der Kwast	MD, FRCSC	Theodorus.vanderKwast@uhn.ca	Cancer Clinical Research Unit (CCRU), Princess Margaret Cancer Centre
Martin		Gleave	MD, FRCSC	m.gleave@ubc.ca	Department of Urologic Sciences, The Vancouver Prostate Centre and University of British Columbia
Ladan		Fazli	MD, FRCSC	lfazli@prostatecentre.com	The Vancouver Prostate Centre and University of British Columbia
